# Multi-omics analysis reveals the panoramic picture of necroptosis-related regulators in pan-cancer

**DOI:** 10.18632/aging.204124

**Published:** 2022-06-21

**Authors:** Guanghao Li, Xiaoxuan Wang, Yongheng Liu, Huikai Li, Han Mu, Yanting Zhang, Qiang Li

**Affiliations:** 1 Tianjin Medical University Cancer Institute and Hospital, National Clinical Research Center for Cancer, Key Laboratory of Cancer Prevention and Therapy, Tianjin’s Clinical Research Center for Cancer, Tianjin 300060, China; 2 Department of Hepatobiliary Cancer, Tianjin Medical University Cancer Institute and Hospital, National Clinical Research Center for Cancer, Tianjin 300060, China; 3 Department of Lymphoma, Tianjin Medical University Cancer Institute and Hospital, National Clinical Research Center for Cancer, Sino-US Center for Lymphoma and Leukemia Research, Tianjin 300060, China

**Keywords:** necroptosis, pan-cancer, genomics, methylation, anti-tumor immunity

## Abstract

Background: Unlike apoptosis, necroptosis is a tightly regulated form of programmed cell death (PCD) that occurs in a caspase-independent manner and is mainly triggered by receptor-interacting serine/threonine-protein kinases RIPK1 and RIPK3 and the RIPK3 substrate mixed-lineage kinase domain-like protein (MLKL). A growing body of evidence has documented that necroptosis, as a novel therapeutic strategy to overcome apoptosis resistance, has potential pro- or anti-tumoral effects in tumorigenesis, metastasis, and immunosurveillance. However, comprehensive multi-omics studies on regulators of necroptosis from a pan-cancer perspective are lacking.

Methods: In the present study, a pan-cancer multi-omics analysis of necroptosis-related regulators was performed by integrating over 10,000 multi-dimensional cancer genomic data across 33 cancer types from TCGA, 481 small-molecule drug response data from CTRP, and normal tissue data from GTEx. Pan-cancer pathway-level analyses of necroptosis were conducted by gene set variation analysis (GSVA), including differential expression, clinical relevance, immune cell infiltration, and regulation of cancer-related pathways.

Results: Genomic alterations and abnormal epigenetic modifications were associated with dysregulated gene expression levels of necroptosis-related regulators. Changes in the gene expression levels of necroptosis-related regulators significantly influenced cancer progression, intratumoral heterogeneity, alterations in the immunological condition, and regulation of cancer marker-related pathways. These changes, in turn, caused differences in potential drug sensitivity and the prognosis of patients.

Conclusion: Necroptosis-related regulators are expected to become novel biomarkers of prognosis and provide a fresh perspective on cancer diagnosis and treatment.

## INTRODUCTION

Although evasion of cell death is a prominent hallmark of cancer, various cellular stress signals can lead to cell death in tumors, such as hypoxia, chemotherapy, immune response, and an environment devoid of essential nutrients [1]. Cell death processes are mainly involved in two mechanisms: caspase-dependent and caspase-independent. Necroptosis, a novel form of programmed cell death [2], is different from apoptosis in that it is often caused by acute cell damage [3]. In contrast to apoptosis, necrosis is characterized by rapid cytoplasmic swelling, increased cell volume, and plasma membrane rupture, resulting in the release of intracellular contents, triggering marked inflammatory responses, and activating the immune system [4, 5]. Necroptosis can be triggered by activation of upstream cell death receptors, the tumor necrosis factor (TNF) receptor superfamily, Toll-like receptors (TLRs), T-cell receptors, interferon receptors, FAS (CD95), virus infection sensor, and some drugs [6–9]. TNF-α-triggered necroptosis is mediated by receptor-interacting serine/threonine-protein kinase 1 (RIPK1), RIPK3, and downstream initiator pseudokinase mixed lineage kinase domain-like protein (MLKL) [3, 10–13]. Upon stimulation by TNFα, RIPK1 is recruited to the cytoplasmic membrane and forms a complex with Fas-associated death domain protein (FADD) and RIPK3 [14]. After that, RIPK3 activated by RIPK1 recruits and phosphorylates MLKL, resulting in the oligomerization and translocation of MLKL [15]. Then, trafficking of active MLKL from the cytoplasm to the cytomembrane, calcium influx-mediated channel formation, disruption of plasma membrane integrity, and necrosis-like cell death occur [16].

Necroptosis could function as a double-edged sword in the development and progression of cancer [17–20]. While necroptosis elicits robust adaptive immune responses and may trigger enhanced anti-tumor immune surveillance and immunotherapy, the consequent inflammatory responses could also promote tumor development and cancer progression directly or indirectly [21, 22]. Necroptosis can mediate resistance to sorafenib and promote tumor metastasis, and it may serve as an indicator of prognosis in hepatocellular carcinoma [23–25]. The induction of necroptosis is a promising alternative therapy for killing cancer cells. The antitumor effects mediated by the RIP1-HAT1-SIRT complex have been reported [26]. Therefore, understanding the expression patterns, potential molecular mechanisms, functional roles, and prognostic impact of necroptosis-related regulators in tumor formation and progression is vital. However, only a few studies have comprehensively characterized necroptosis-related regulators from a pan-cancer perspective.

In the present study, we systematically and comprehensively explored necroptosis-related regulators by using pan-cancer multi-omics data from The Cancer Genome Atlas (TCGA). We found that genomic alterations and epigenetic modifications of necroptosis-related regulators could lead to their abnormal expression and affect the prognosis of cancer patients. Furthermore, expression levels of necroptosis-related regulators were significantly correlated with molecular subtypes, clinicopathologic stage, immune subtypes, regulation of cancer-related pathways, sensitivity to anticancer drugs, and prognostic outcomes in various cancers. Necroptosis-related regulators may be potential therapeutic targets of multiple cancers and improve the landscape of current cancer treatment.

## RESULTS

### mRNA expression and prognostic value of necroptosis-related regulators

**Figure 1:**
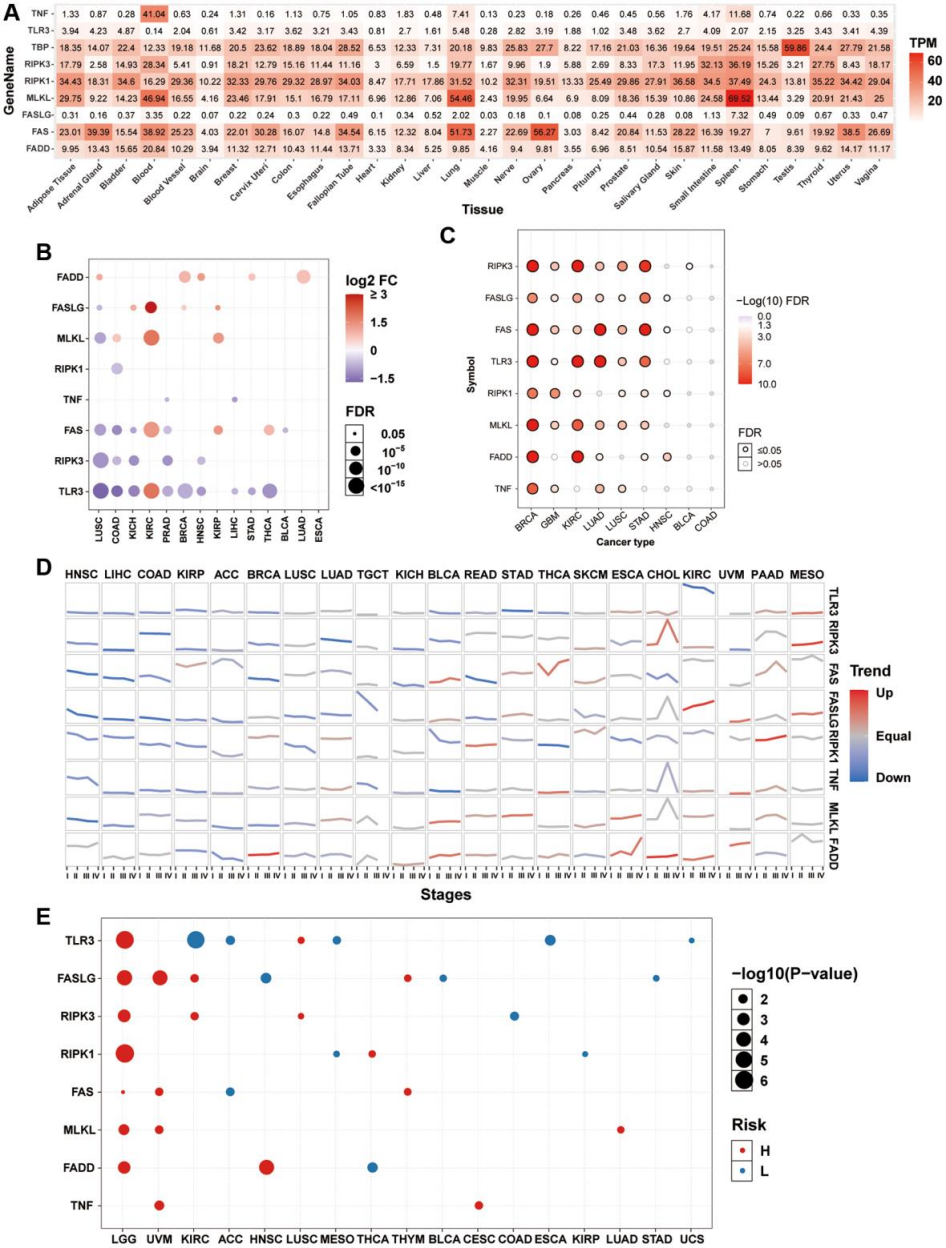
**mRNA expression and survival analysis of necroptosis-related regulators.** (**A**) mRNA expression of necroptosis-related regulators in the GTEx normal tissues. (**B**) Differential mRNA expression of necroptosis-related regulators between paired tumor and normal tissue. The size of dots is positively correlated with the FDR significance. The color of the bubble represents the fold change between tumor vs. normal. The bubble was filtered by the fold change (FC>2) and significance (FDR ≤ 0.05). (**C**) Subtype-related changes in gene expression of necroptosis-related regulators. The bubble color from white to red represents the FDR significance, and the bubble size is positively correlated with the FDR significance. The black outline border of bubble indicates FDR ≤ 0.05. (**D**) The trend of the gene expression of necroptosis-related regulators from stage I to stage IV in different cancers. The blue trend line and red trend line represent fall and rise tendency, respectively. (**E**) Survival analysis of necroptosis-related regulators in different cancers. The bubble color from blue to red represents the hazard ratio from low to high, bubble size is positively correlated with the Cox *P*-value significance.

To explore the gene expression of necroptosis-related regulators in normal tissues, we extracted mRNA data of necroptosis-related regulators from the GTEx dataset. In all normal tissues, the expression of TNF, TLR3, and FASLG was lower than that of FADD, FAS, MLKL, RIPK1, and RIPK3. The expression levels of TNF in the blood, MLKL in the blood, lung, and spleen, and FAS in the lung and ovary were relatively higher than their expression levels in the normal tissues (Figure 1A). Differential expression analysis between tumor-normal paired samples from 14 TCGA cancer types showed that the expression levels of most necroptosis-related regulators were dysregulated in various tumor types. The mRNA expression levels of FADD in lung squamous cell carcinoma (LUSC), breast invasive carcinoma (BRCA), head and neck squamous cell carcinoma (HNSC), stomach adenocarcinoma (STAD), and lung adenocarcinoma (LUAD); FASLG in kidney chromophobe (KICH), kidney renal clear cell carcinoma (KIRC), BRCA, and KIRP; MLKL in colon adenocarcinoma (COAD), KIRC, and KIRP; FAS in KIRC, KIRP, and thyroid carcinoma (THCA); and TLR3 in KIRC were significantly upregulated. However, the mRNA expression levels of FASLG in LUSC; MLKL in LUSC; RIPK1 in COAD; TNF in prostate adenocarcinoma (PRAD) and liver hepatocellular carcinoma (LIHC); FAS in LUSC, COAD, KICH, PRAD, and bladder urothelial carcinoma (BLCA); RIPK3 in LUSC, COAD, KICH, PRAD, and HNSC; and TLR3 in LUSC, COAD, KICH, PRAD, BRCA, HNSC, LIHC, STAD, and THCA were significantly downregulated (Figure 1B). The mRNA expression of the same necroptosis-related regulator was upregulated in one cancer type but downregulated in another cancer type, such as TLR3 in KIRC (Supplementary Figure 1A) and LUSC (Supplementary Figure 1B). Almost all necroptosis-related regulators displayed a subtype-specific expression pattern in BRCA, GBM, KIRC, LUAD, LUSC, and STAD (Figure 1C). For example, the expression of RIPK3 was higher in the luminal subtype, especially the luminal A subtype (Supplementary Figure 1C), while the expression of MLKL was higher in the her2 and basal subtypes, especially in the basal subtype (Supplementary Figure 1D). Expression trend analysis in 21 TCGA cancer types with clinicopathologic information revealed that most necroptosis-related regulators are downregulated progressively with advancing stage in many tumor types. In contrast, only a few necroptosis-related regulators showed exactly the opposite trend, such as FASLG in KIRC and RIPK1 in PAAD (Figure 1D). In addition, overall survival analysis showed that the expression of many necroptosis-related regulators had different effects on the prognosis of cancer patients. The high expression of TLR3 in lower grade glioma (LGG) and LUSC; FASLG in LGG, UVM, KIRC, and THYM; RIPK3 in LGG, KIRC, and LUSC; RIPK1 in LGG and THCA; FAS in LGG, UVM, and THYM; MLKL in LGG, UVM, and LUAD; FADD in LGG and HNSC; and TNF in UVM and CESC was associated with poor survival. In contrast, the high expression of TLR3 in KIRC, ACC, MESO, esophageal carcinoma (ESCA), and uterine carcinosarcoma (UCS); FASLG in HNSC, BLCA, and STAD; RIPK3 in COAD; RIPK1 in MESO and KIRP; FAS in ACC; and FADD in THCA was associated with good survival (Figure 1E). The mRNA expression of the same necroptosis-related regulator had different effects on the prognosis of different cancer types. For example, the high expression of TLR3 was associated with good survival in KIRC (Supplementary Figure 1E) but poor survival in LUSC (Supplementary Figure 1F). These results indicated that the abnormal expression of necroptosis-related regulators contributes to tumorigenesis, cancer progression, and intratumoral heterogeneity.

### Methylation analysis of necroptosis-related regulators

**Figure 2:**
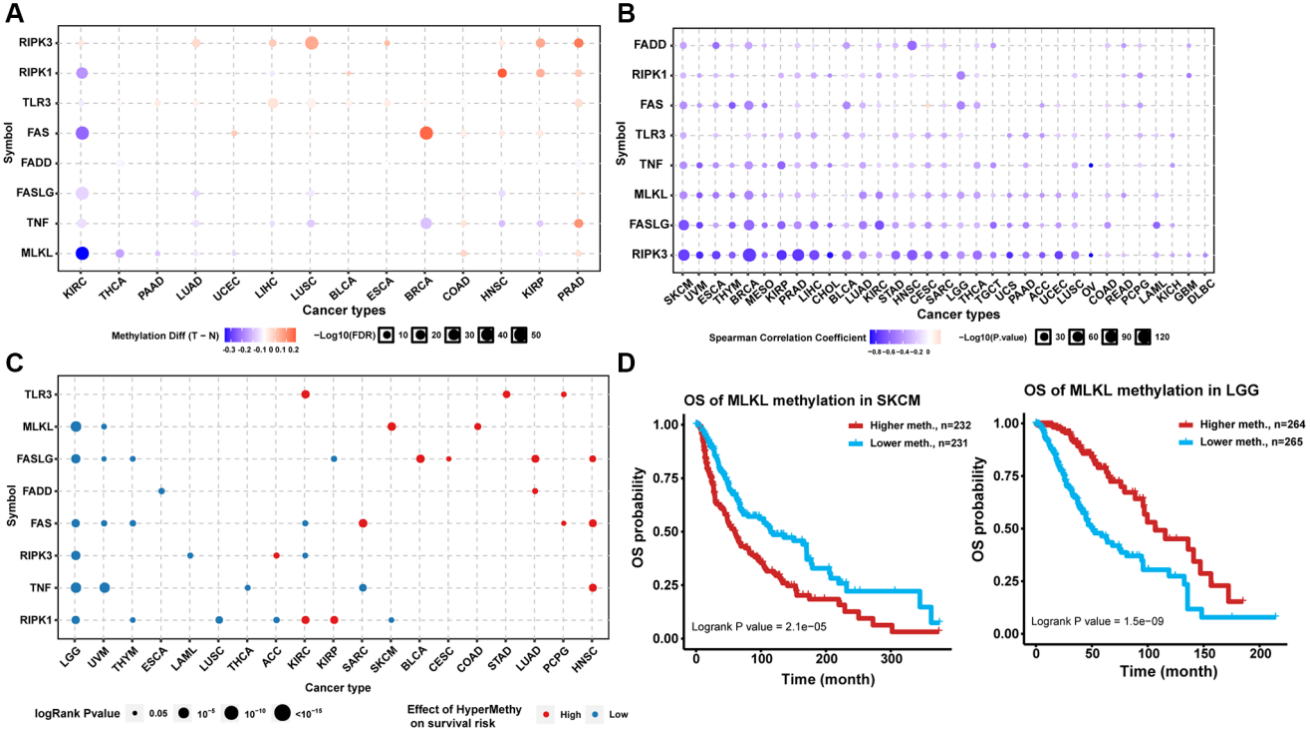
**Methylation analysis of necroptosis-related regulators.** (**A**) Differential methylation status of necroptosis-related regulators between normal and tumor tissues in different cancers. The red bubble and blue bubble represent hypermethylation and hypomethylation in tumors, respectively. The bubble size is positively correlated with the FDR significance, and the bubble was filtered by FDR significance (FDR ≤ 0.05). (**B**) Correlation between methylation level and mRNA expression. The blue bubble and red bubble represent negative and positive correlations, respectively. The bubble size is positively correlated with the significance of FDR. (**C**) Methylation survival analysis of necroptosis-related regulators in different cancers. The bubble color from blue to red represents the hazard ratio from low to high, and the bubble size is positively correlated with the Cox *P*-value significance. (**D**) Kaplan-Meier curves between high and low methylation groups of MLKL in SKCM and LGG.

Analysis of the methylation status of necroptosis-related regulators between tumor and normal samples from 14 TCGA cancer types showed that RIPK3 and TLR3 exhibited DNA hypermethylation in multiple tumors. Most necroptosis-related regulators exhibited DNA hypomethylation in KIRC while exhibiting DNA hypermethylation in PRAD (Figure 2A). Correlation analysis indicated that the expression levels of almost all necroptosis-related regulators were associated negatively with the degree of DNA methylation in pan-cancer samples (Figure 2B). Analysis of prognosis indicated that the hypermethylation of TLR3 in KIRC, STAD, and PCPG; MLKL in skin cutaneous melanoma (SKCM) and COAD, FASLG in BLCA, CESC, LUAD, and HNSC; FADD in LUAD; FAS in SARC, PCPG, and HNSC; RIPK3 in ACC; TNF in HNSC; RIPK1 in KIRC and KIRP was associated with poor prognosis, while the hypomethylation of MLKL in LGG and UVM; FASLG in LGG, UVM, THYM, and KIRP; FADD in ESCA; FAS in LGG, UVM, THYM, and KIRC; RIPK3 in LGG, LAML, and KIRC; TNF in LGG, UVM, THCA, and SARC; and RIPK1 in LGG, THYM, LUSC, ACC, and SKCM was associated with poor prognosis (Figure 2C). The hypermethylation of the same necroptosis-related regulator could have different effects on prognosis in different cancer types, such as MLKL in LGG and SKCM (Figure 2D). These results suggested that abnormal epigenetic modification patterns of necroptosis-related regulators existed in multiple tumors and could affect gene expression and prognosis of cancer patients.

### Mutational landscape of necroptosis-related regulators

**Figure 3:**
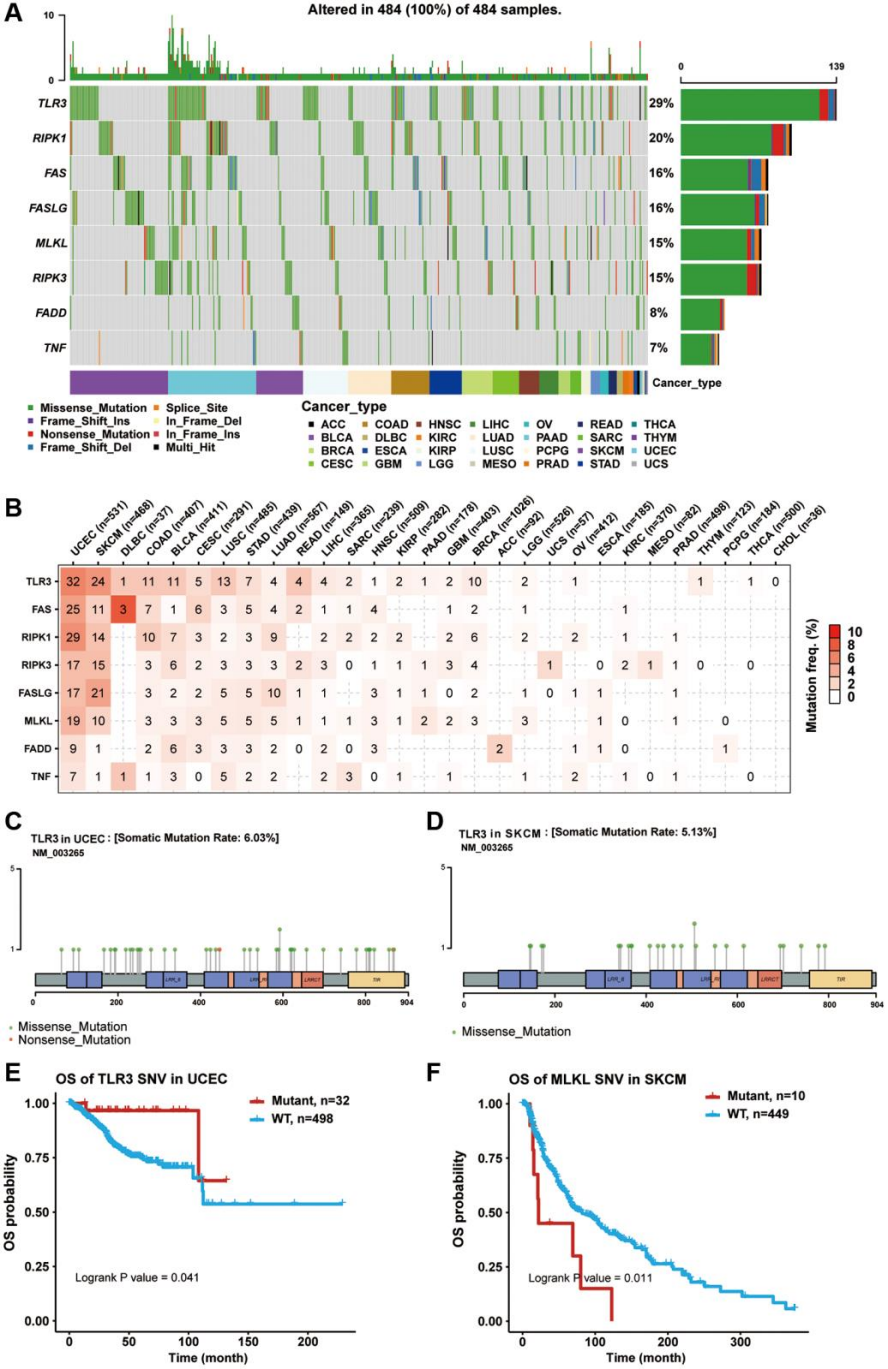
**Single nucleotide variation (SNV) analysis of necroptosis-related regulators.** (**A**) Oncoplot showing the SNV frequency distribution of necroptosis-related regulators in pan-cancer. Side barplot and top barplot show the number of variants in each gene and each sample, respectively. (**B**) The percentage heatmap showed the SNV frequency of necroptosis-related regulators in specific cancer type. The color depth is positively correlated with mutate frequency. The number in each cell represents the number of mutated samples in specific cancer. The 0 and blank in the cell indicate there is no mutation in specific gene coding region and all regions of a specific gene, respectively. (**C**) Lollipop plot showing the mutation site, type and frequency of TLR3 in UCEC. (**D**) Lollipop plot showing the mutation site, type and frequency of TLR3 in SKCM. (**E**) Kaplan-Meier curve between WT and Mutant groups of TLR3 in UCEC. (**F**) Kaplan-Meier curve between WT and Mutant groups of MLKL in SKCM.

Somatic mutations in cancer genomes are related to the occurrence and development of cancer. Thus, we explored SNP data of necroptosis-related regulators in pan-cancer samples. A waterfall plot showed that the mutation frequencies of TLR3, RIPK1, FAS, FASLG, MLKL, RIPK3, FADD, and TNF were 29%, 20%, 16%, 16%, 15%, 15%, 8%, and 7%, respectively (Figure 3A). In addition, a single nucleotide variation (SNV) summary plot showed that the most abundant SNV class (base substitution) was C>T, and the variant classification and type were mainly missense mutation and SNP, respectively (Supplementary Figure 2). In the SNV percentage analysis, we found that the number of samples in which the necroptosis-related regulators had deleterious mutations was greater in uterine corpus endometrial carcinoma (UCEC) and SKCM, especially TLR3, with the highest mutation frequency among all cancer types (Figure 3B–3D). Survival analysis found that the mutations of multiple necroptosis-related regulators could cause different effects on the overall prognosis of cancer patients (data not shown). For example, TLR3 mutation was associated with poor survival in UCEC (Figure 3E), while MLKL mutation was associated with better survival in SKCM (Figure 3F). These results indicated that necroptosis-related regulators were particularly prone to undergo mutation in tumors, and their mutations could also impact the clinical outcomes of cancer patients.

### Copy number variation (CNV) of necroptosis-related regulators

**Figure 4:**
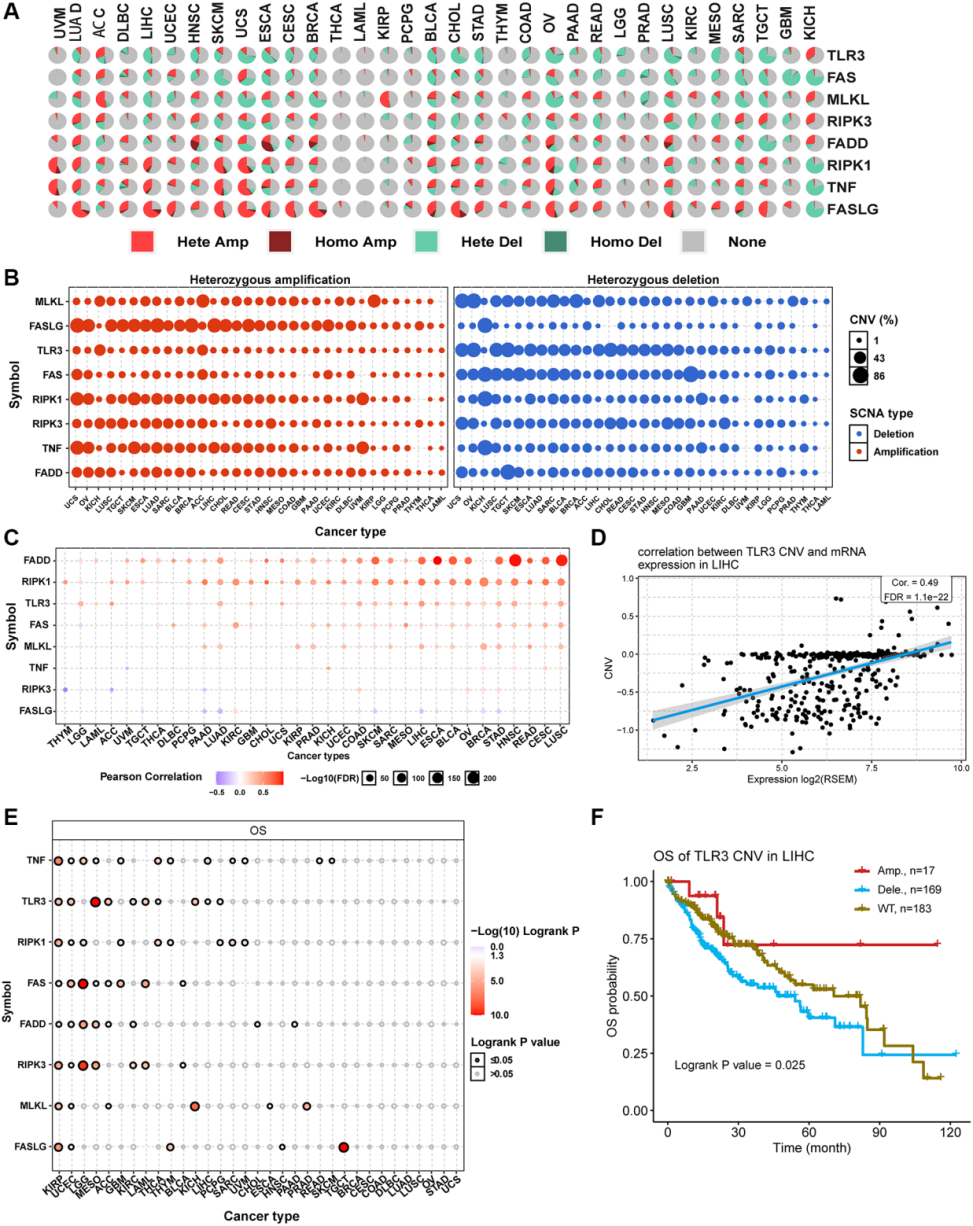
**Copy number variation (CNV) analysis of necroptosis-related regulators.** (**A**) CNV pie plot showing the constitute of Heterozygous/Homozygous CNV of necroptosis-related regulators in different cancers. Hete Amp, heterozygous amplification; Homo Amp, homozygous amplification; Hete Del, heterozygous deletion; Homo Del, homozygous deletion; None, no CNV. (**B**) Heterozygous CNV plot showing the percentage of heterozygous amplification (red bubble) and deletion (blue bubble) of necroptosis-related regulators in different cancers. The bubble size is positively correlated with percentage. (**C**) The association between CNV level and mRNA expression of necroptosis-related regulators in different cancers. Blue bubble and red bubble represent negative and positive correlations, respectively. The deeper the color, the stronger the correlation. Bubble size is positively correlated with the FDR significance. (**D**) Scatter plot showing the correlation between TLR3 CNV and its mRNA expression in LIHC. (**E**) CNV survival analysis of necroptosis-related regulators in different cancers. (**F**) Kaplan-Meier curve showing the survival difference between different CNV types and wild type of TLR3 in LIHC.

CNV is a prevalent and important hallmark of many cancers. For this reason, we investigated the CNV changes of necroptosis-related regulators. The CNV pie plot shows that most necroptosis-related regulators had many copy number amplifications and deletions in most cancer types except for THCA and LAML (Figure 4A). FASLG in LIHC had the highest relative percentage of total amplification, while FAS in GBM had the highest relative percentage of total deletions (Supplementary Figure 3A). In addition, heterozygous CNV analysis showed that almost all necroptosis-related regulators had heterozygous amplification and deletion among all cancer types (Figure 4B). Meanwhile, homozygous CNV analysis showed that FADD in 22 cancer types, FASLG in 25 cancer types, RIPK1 in 22 cancer types, TLR3 in 15 cancer types, TNF in 23 cancer types, FAS in 15 cancer types, MLKL in 12 cancer types, and RIPK3 in 17 cancer types had homozygous amplifications. In comparison, FADD in 12 cancer types, FASLG in 3 cancer types, RIPK1 in 19 cancer types, TLR3 in 25 cancer types, TNF in 11 cancer types, FAS in 22 cancer types, MLKL in 15 cancer types, and RIPK3 in 12 cancer types had homozygous deletions (Supplementary Figure 3B). Correlation analysis indicated that the mRNA expression levels of necroptosis-related regulators were positively correlated with their copy number levels in most cancers, such as TLR3 in LIHC (Figure 4C and 4D). However, the mRNA expression levels of FASLG were negatively correlated with its copy number levels in multiple tumors, such as in HCSC (Supplementary Figure 3C). Survival analysis showed that CNVs of TNF in 22 cancer types, TLR3 in 10 cancer types, RIPK1 in 9 cancer types, FAS in 8 cancer types, FADD in 8 cancer types, RIPK3 in 7 cancer types, MLKL in 6 cancer types, and FASLG in 5 cancer types were significantly associated with overall prognosis (Figure 4E). For example, the copy number deletions of TLR3 in LIHC (Figure 4F) and FASLG in HNSC (Supplementary Figure 3D) were associated with poor prognosis compared with normal copy number and copy number amplifications. The CNV of all necroptosis-related regulators could affect the prognosis of KIRC and UCEC patients (Figure 4E). These results indicated that the different types of CNVs of necroptosis-related regulators were widespread in many cancer types and could affect mRNA expression and the prognosis of cancer patients.

### miRNA regulation of necroptosis-related regulators

**Figure 5:**
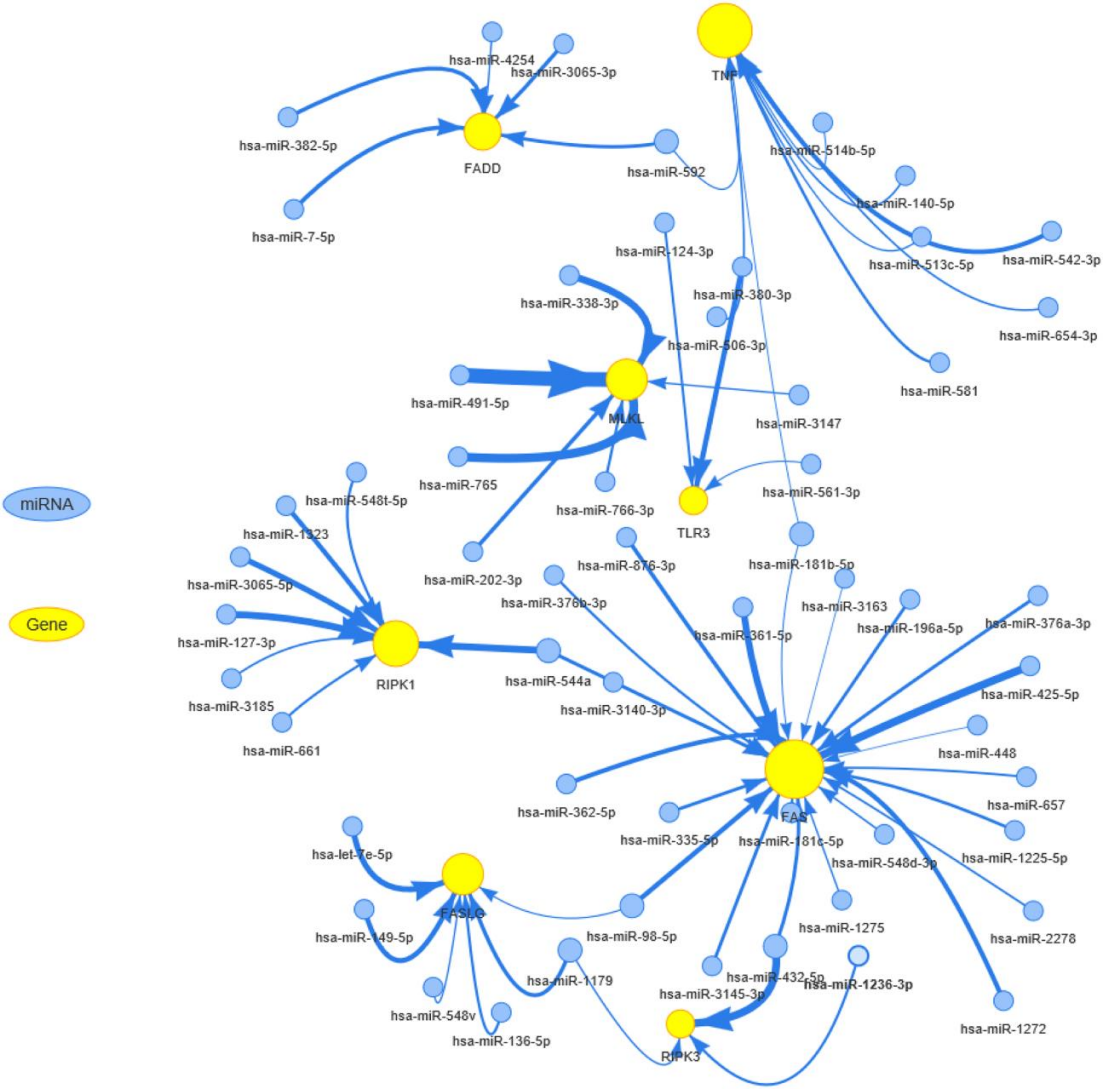
**The miRNA regulation network of necroptosis-related regulators.** The connection between miRNA and gene suggests the miRNA can regulate the gene. The node size is positively correlated with the node's degree, and the width of the line is decided by the absolute value of the correlation coefficient.

To clarify which miRNA could regulate the mRNA expression of necroptosis-related regulators, we used the R package visNetwork to construct the miRNA-gene regulation network (Figure 5). In this network, the node size of genes and edge width positively correlated with the number of related miRNAs and correlation coefficient, respectively. TNF and FAS were negatively regulated by more miRNAs. Each necroptosis-related regulator could be regulated by multiple miRNAs. For example, both RIPK1 and FAS could be negatively regulated by hsa-miR-554a, while hsa-miR-181b-5p could negatively regulate both FAS and TNF. These results indicated that complex miRNA regulatory networks regulated the mRNA expression levels of necroptosis-related regulators.

### Pathway activity analysis

**Figure 6:**
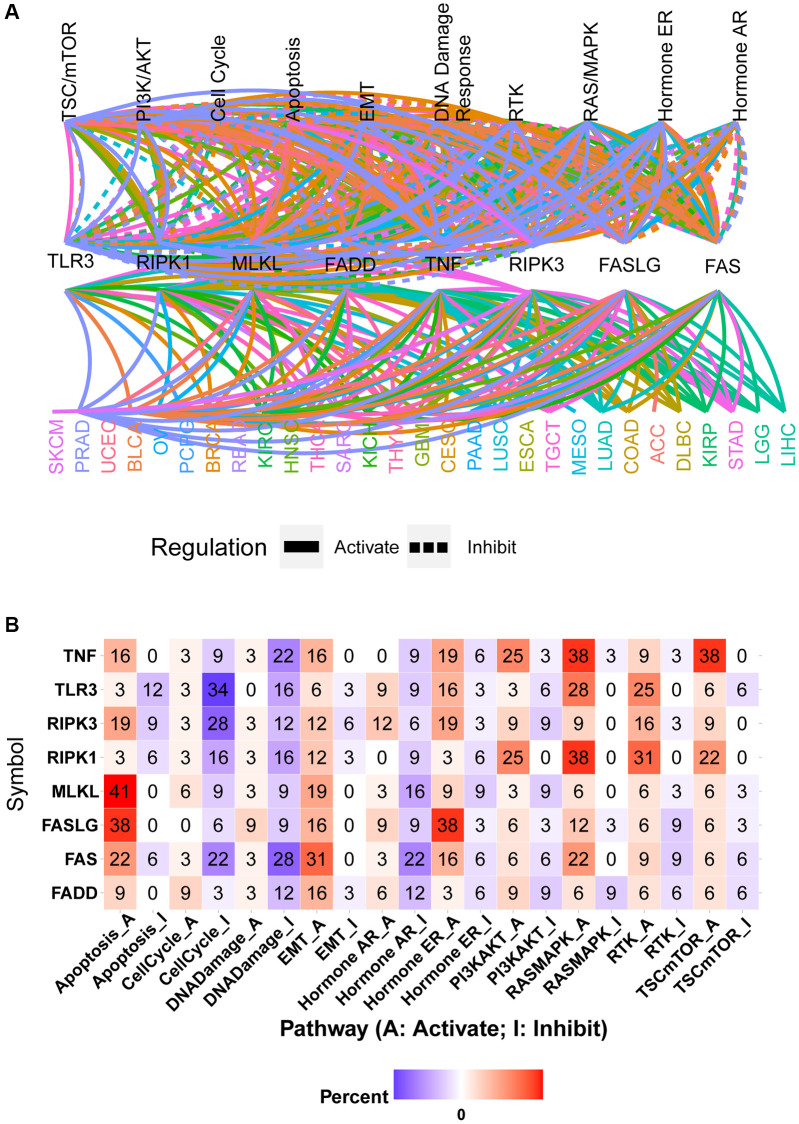
**Pathway activity analysis of necroptosis-related regulators.** (**A**) Gene-pathway network showed the regulatory relationship between necroptosis-related regulators and cancer pathways in pan-cancer. Different colors represent different cancer types. (**B**) The heatmap showed the percentage of cancer types in which the specific necroptosis-related regulator has an effect (FDR ≤ 0.05) on the specific pathway in pan-cancer.

The global regulation network showed that the mRNA expression of necroptosis-related regulators had close associations with the activity of cancer-related pathways in pan-cancer samples (Figure 6A). The same necroptosis-related regulator could produce different regulatory effects on the same pathway in different cancer types. For example, high expression of FAS could activate the apoptosis pathway in PAAD while inhibiting the activation of the apoptosis pathway in KIRP (Supplementary Figure 4A and 4B). The pathway heatmap and pie chart show the percentage of cancer types (cancer types/32 ^*^100%) in which necroptosis-related regulators affected the specific pathway (Figure 6B and Supplementary Figure 4C). The apoptosis pathway was mainly activated by TNF (16% activation vs. 0% inhibition), RIPK3 (19% activation vs. 9% inhibition), MLKL (41% activation vs. 0% inhibition), FASLG (38% activation vs. 0% inhibition), FAS (22% activation vs. 6% inhibition), and FADD (9% activation vs. 0% inhibition) and inhibited by TLR3 (3% activation vs. 12% inhibition) and RIPK1 (3% activation vs. 6% inhibition). Similarly, the cell cycle pathway was mainly activated by FADD and inhibited by TNF, TLR3, RIPK3, RIPK1, MLKL, FASLG, and FAS. Except for FASLG, all other necroptosis-related regulators could inhibit the DNA damage pathway. All necroptosis-related regulators could activate the EMT pathway. The hormone AR pathway was mainly activated by RIPK3 and inhibited by TNF, RIPK1, MLKL, FAS, and FADD. The hormone ER pathway was mainly activated by TNF, RIPK3, TLR3, FASLG, and FAS and inhibited by RIPK1 and FADD. The PI3K/AKT pathway was mainly activated by TNF, RIPK1, and FASLG and inhibited by TLR3 and MLKL. Except for being inhibited by FADD, the RAS/MAPK pathway could be activated by all other necroptosis-related regulators. The RTK pathway was mainly activated by TNF, TLR3, RIPK3, RIPK1, and MLKL and inhibited by FASLG. The TSC/mTOR pathway could be activated by TNF, RIPK3, RIPK1, MLKL, and FASLG (Figure 6B). These results indicated that necroptosis-related regulators contribute to the activation or inhibition of cancer-related pathways.

### Immune subtype and drug sensitivity analysis

**Figure 7:**
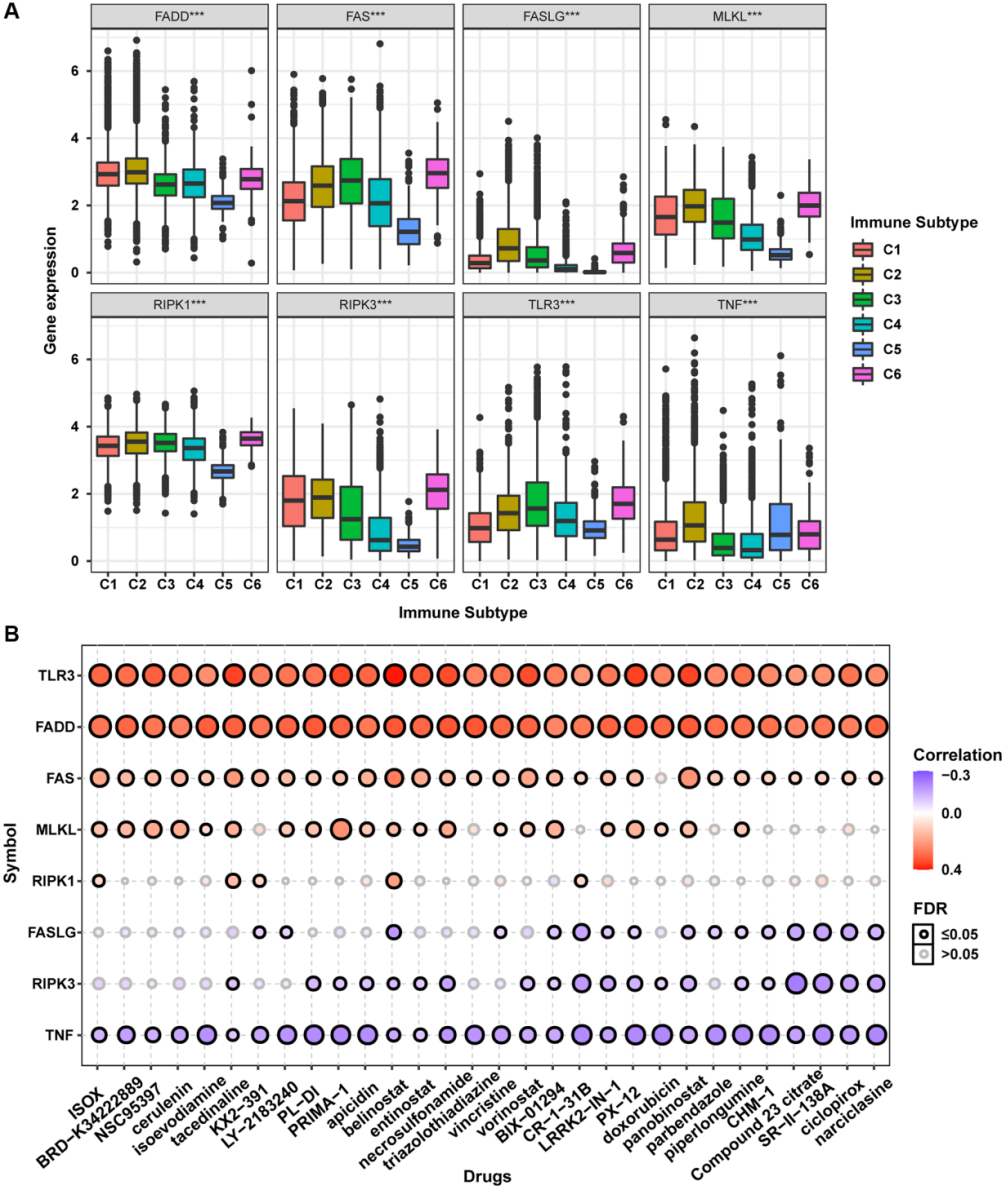
**Immune subtype and drug sensitivity analysis of necroptosis-related regulators**. (**A**) Expression differences of necroptosis-related regulators between six pan-cancer immune subtypes. (**B**) Bubble plot showing the correlation between drug sensitivity (IC50) and gene expression level of necroptosis-related regulators in CTRP database. Positive correlation (red bubble) indicates one gene with high expression was resistant to a drug, while negative correlation (blue bubble) indicates one gene with high expression was sensitive to a drug. The color depth and size of bubble are positively correlated with the correlation coefficient and the FDR significance, respectively. Black outline border indicates FDR ≤ 0.05.

The accumulation of extensive genomic alterations might affect the immune response of cancer patients and sensitivity to anticancer drugs [27, 28]. Immune subtype analysis to determine whether the necroptosis-related regulators would affect cancer patients’ immune status showed that all necroptosis-related regulators were differentially expressed in different immune subtypes (Figure 7A). Except for TNF, all other necroptosis-related regulators were lowly expressed in the C5 subtype and highly expressed in the C6 subtype. Drug sensitivity analysis showed that the mRNA expression levels of TLR3, FADD, FAS, MLKL, and RIPK1 were mainly negatively correlated with drug sensitivity. In contrast, FASLG, RIPK3, and TNF mRNA expression levels were mainly positively correlated with drug sensitivity (Figure 7B). The mRNA expression levels of TLR3 and FADD were negatively correlated with the sensitivity of all top 30 ranked drugs (positive correlation with IC50). Except for doxorubicin, the mRNA expression of FAS was negatively correlated with sensitivity to all other 29 anticancer drugs (positive correlation with IC50). The mRNA expression of MLKL was negatively correlated with sensitivity to 21 anticancer drugs except for KX2-391, necrosulfonamide, CR-1-31B, panobinostat, piperlongumine, CHM-1, Compound 23 citrate, SR-II-138A, ciclopirox, and narciclasine (positive correlation with IC50). The mRNA expression of RIPK1 was negatively correlated with sensitivity to ISOX, isoevodiamine, tacedinaline, belinostat, and CR-1-31B. The mRNA expression of FASLG was positively correlated with sensitivity to tacedinaline, LY-2183240, belinostat, triazolothiadiazine, BIX-01294, CR-1-31B, LRRK2-IN-1, PX-12, doxorubicin, panobinostat, parbendazole, piperlongumine, CHM-1, Compound 23 citrate, SR-II-138A, ciclopirox, and narciclasine (negative correlation with IC50). The mRNA expression of RIPK3 was positively correlated with sensitivity to 20 anticancer drugs except for ISOX, BRD-K34222889, NSC95397, cerulenin, isoevodiamine, KX2-391, LY-2183240, triazolothiadiazine, vincristine, and piperlongumine (negative correlation with IC50). The mRNA expression of TNF was positively correlated with sensitivity to all top 30 ranked drugs (negative correlation with IC50). The mRNA expression levels of all necroptosis-related regulators were associated with sensitivity to belinostat. These results indicated that the dysregulated expression of necroptosis-related regulators is closely associated with the tumor immune microenvironment and affects the response to anticancer therapy.

### GSVA

**Figure 8:**
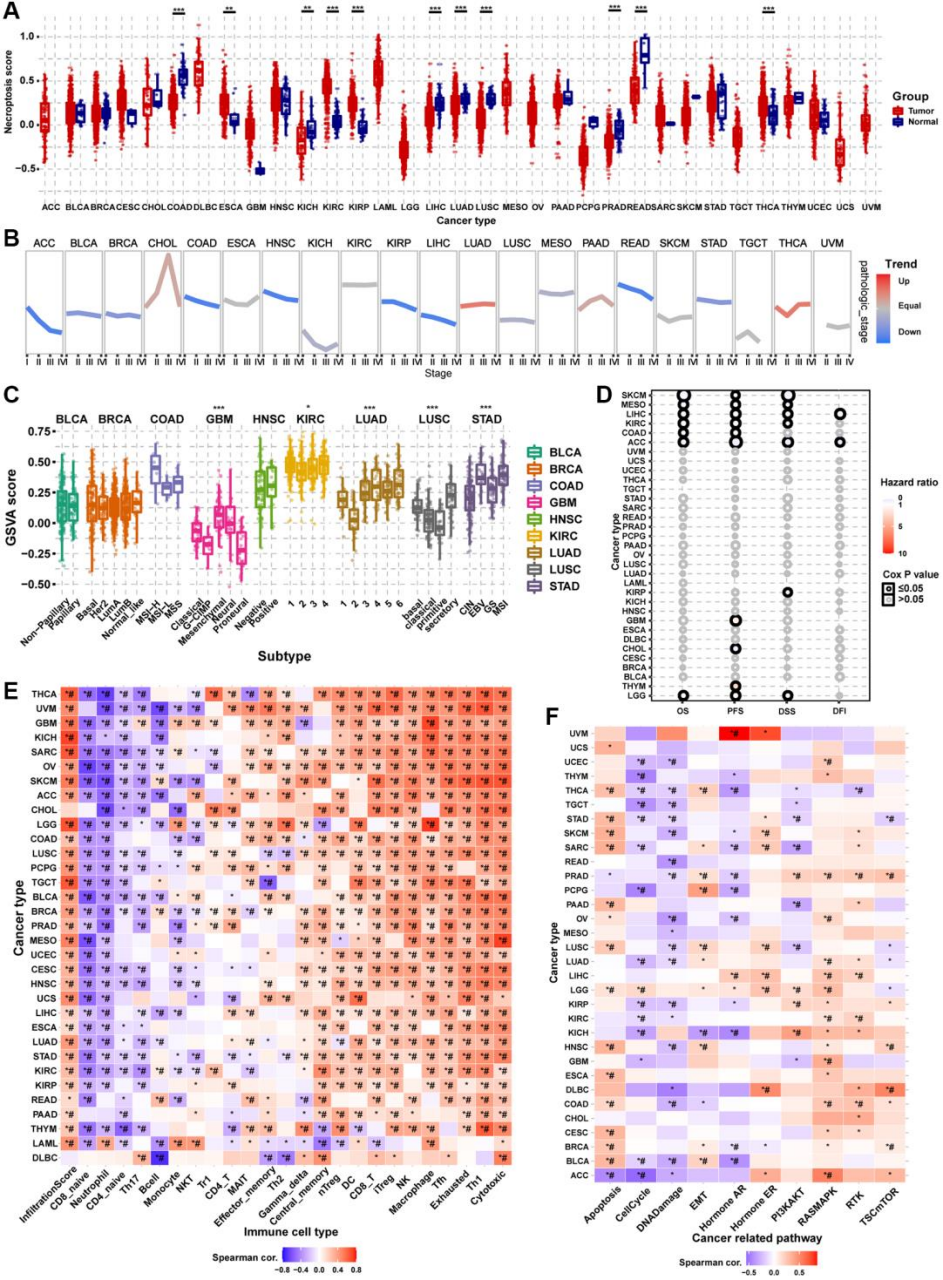
**Gene Set Enrichment Analysis (GSVA) analysis of necroptosis-related regulators.** (**A**) The differences of necroptosis score between tumor and normal samples in pan-cancer. The necroptosis score represents the integrated level of the expression of necroptosis-related regulators, which is positively correlated with gene expression. (**B**) The trend of the necroptosis score from stage I to stage IV in different cancers. The blue trend line and red trend line represent fall and rise tendency, respectively. (**C**) Box plot showing the differences of necroptosis score between different cancer subtypes. (**D**) Survival analysis of necroptosis score in different cancer types, including overall survival (OS), progression-free survival (PFS), disease-specific survival (DSS), and disease-free survival (DFI). (**E**) Heatmap showing the correlation between the necroptosis score and immune cell infiltration in different cancer types. ^*^P ≤ 0.05; ^#^FDR ≤ 0.05. (**F**) Heatmap showing the correlation between the necroptosis score and pathway activity in different cancer types. ^*^P ≤ 0.05; ^#^FDR ≤ 0.05.

Differential expression analysis showed that the necroptosis score was dysregulated in multiple tumor types. In COAD, KICH, LIHC, LUAD, LUSC, PRAD, and rectum adenocarcinoma (READ), the necroptosis score of tumor tissue was lower than that of the normal tissue. However, the necroptosis score was higher in tumor tissues in ESCA, KIRC, KIRP, and THCA (Figure 8A). Pathologic stage trend analysis showed that the necroptosis score was downregulated progressively with advancing stage in many tumor types, including ACC, BLCA, BRCA, COAD, HNSC, KIRP, LIHC, READ, and STAD (Figure 8B). Subtype analysis showed that the necroptosis scores significantly differed with the tumor subtype, including GBM, KIRC, LUAD, LUSC, and STAD (Figure 8C). Survival analysis showed that the necroptosis score could affect the OS of SKCM, MESO, LIHC, KIRC, COAD, ACC, and LGG; the PFS of SKCM, MESO, LIHC, KIRC, COAD, ACC, GBM, CHOL, THYM, and LGG; the DSS of SKCM, MESO, LIHC, KIRC, ACC, KIRP, and LGG; and the DFI of LIHC and ACC (Figure 8D). The necroptosis score could affect more than one type of survival in some cancer types. For example, patients with higher necroptosis scores had longer OS, PFS, DSS, and PFI in LIHC (Supplementary Figure 5). The results of immune infiltration analysis were generally consistent across the different cancer types (Figure 8E). The necroptosis score was positively correlated with the abundance of Tr1, central memory, gamma delta, NK, Th2, CD4_T, DC, nTreg, Tfh, MAIT, macrophage, cytotoxic, effector memory, iTreg, CD8_T, exhausted and Th1 cells. On the contrary, the necroptosis score was negatively correlated with the abundance of B cell, neutrophil, NKT, CD4_naive, Th17, monocyte, and CD8_naive cells. The necroptosis score of almost all types of cancer was positively correlated with the infiltration score. The pathway activity analysis results to explore the intrinsic interactions between the necroptosis score and canonical cancer-related pathways were also generally consistent across the different cancer types (Figure 8F). In most tumors, the necroptosis score was positively correlated with apoptosis, EMT, hormone ER, RAS/MAPK, RTK, and TSC/mTOR pathway activation. However, the necroptosis score was negatively correlated with the pathway activation of cell cycle, DNA damage, hormone AR, and PI3K/AKT. These results indicated that necroptosis affects tumor progression, subtype heterogeneity, recruitment of immune cells, regulation of oncogenic pathways, and prognosis of cancer patients.

## DISCUSSION

Most tumors are characterized by sustained proliferative signaling and resistance to apoptosis. Therefore, triggering cell death by other mechanisms, such as necroptosis, has become a promising anticancer strategy [29]. Necroptosis is closely related to more aggressive phenotypes and poor prognosis in many cancer types, including lung cancer, colorectal cancer, and breast cancer [30–37]. Thus, studying the role of necroptosis-related regulators in tumorigenesis and cancer progression would facilitate the discovery of clinically relevant therapeutic targets. In this study, we performed a systematic integrative investigation of eight necroptosis-related regulators across 33 cancer types by using pan-cancer multi-omics data. Our results revealed that genomic alterations, abnormal epigenetic modifications, and complex regulation of miRNAs of necroptosis-related regulators led to dysregulated gene expression, which correlated significantly with changes in the immune microenvironment and disruption of hallmark cancer-related pathways. To our knowledge, this is the first report of necroptosis-related regulators from a pan-cancer perspective.

In gene expression and survival analysis, we found that the necroptosis-related regulators exhibited varying degrees of expression dysregulation in multiple cancers, which could affect the progression of clinical stage, subtype heterogeneity, and final outcomes of cancer patients. As a significant differentially expressed gene in 10 cancer types, the low expression of TLR has been demonstrated in breast cancer [38]. In addition, the stable overexpression of TLR3 could inhibit cell proliferation *in vitro* and *in vivo* and correlate with less invasive phenotypes of breast cancer cells [38]. TLR3 was found to be overexpressed in 139 of 189 (73.5%) cases of clear cell renal cell carcinoma and in 6 of 8 lung metastatic clear cell renal cell carcinoma compared to the very low expression in the normal kidney tissue [39]. Kim et al. reported that the high expression of TLR3 was closely related to a high level of neutrophil infiltration and poor survival of patients with lung cancer [40]. Our findings were consistent with the findings of these studies. However, there was also non-conformity between aberrant gene expression and clinical prognosis. For example, the mRNA expression of TLR3 was not different in ESCA; however, the high expression of TLR3 was associated with good survival in ESCA. Thus, we speculated that necroptosis-related regulators might undergo altered genetic modifications in tumor progression.

Epigenetic and genome analysis showed that necroptosis-related regulators had extremely complex genomes with abnormal epigenetic modification patterns, high mutation frequency, and extensive CNVs in pan-cancer samples. These changes mediated the transcriptional dysregulation of necroptosis-related regulators and dramatically altered cancer prognosis. Pre-treatment with 5-AD (a demethylating agent) increases the expression of MLKL and the activation of necroptosis in melanoma cell lines [41]. In addition, FAS carries the third-highest gene mutational frequency in pan-cancer samples, and FAS mutations have been proven to correlate with cancer development and progression in various tumor types, including malignant glioma [42], breast cancer [43], pulmonary adenocarcinomas [44], and cholangiocarcinoma [45]. Copy number loss of the “death receptor” FAS was found in 35% (13/38) of gliomas, which could affect the gliomagenesis and response to therapy [46]. The miRNA-mRNA interaction network analysis showed that the miRNAs could negatively regulate the necroptosis-related regulators, suggesting that they can be used to control cancer progression and metastasis [47–50]. These results indicated that miRNAs can be used to inhibit tumor progression and improve survival in patients with various cancers.

Pathway analysis showed that the necroptosis-related regulators target various cancer-related signaling pathways. Overall, necroptosis-related regulators could activate apoptosis, EMT, hormone ER, RAS/MAPK, RTK, and TSC/mTOR and inhibit cell cycle, DNA damage, and hormone AR pathways, which have been implicated in several cancers [51–56]. Necroptosis is closely associated with immune response and drug sensitivity [57, 58]. Immune subtype analysis showed that the mRNA expression of necroptosis-related regulators differed greatly from the immune subtype. Drug sensitivity analysis allowed screening of potential anticancer drugs specifically targeting the necroptosis-related regulators. Hong et al. reported that the overexpression of miR-204-5p can be involved in tumor immune microenvironment remodeling or reprogramming through the TNF signaling pathway [59]. In addition, as a key necroptosis-associated molecule, TNF is reported to increase the sensitivity of many cancer types to doxorubicin [60–62]. However, the correlation between sensitivity to belinostat and the expression of all eight necroptosis-related regulators has not been reported. Thus, further research into this finding is needed. Finally, we again confirmed that necroptosis is closely related to tumorigenesis, cancer progression, intratumoral heterogeneity, immune-active microenvironment, regulation of cancer-related pathways, and final clinical outcomes through GSVA.

Taken together, our comprehensive pan-cancer analysis of necroptosis-related regulators will help uncover their potential roles in cancer progression and provide new clues for the accurate diagnosis and treatment of various cancers.

## MATERIALS AND METHODS

### Data sources

The necroptosis gene list was obtained from the MSigDB gene set “GOBP_NECROPTOTIC_SIGNALING_PATHWAY” (http://www.gsea-msigdb.org/). TPM (Transcripts per Million) normalized RNAseq data (data release version 7.0) were downloaded from the Genotype-Tissue Expression (GTEx) web portal (https://commonfund.nih.gov/GTEx/) to explore the gene expression of necroptosis-related regulators in different normal tissues obtained from healthy individuals. The dataset was composed of 11,688 samples containing the expression profiles of 56,202 genes from 30 organs (53 tissues), which were donated by 714 healthy individuals. Multi-omics pan-cancer datasets were acquired from TCGA (The Cancer Genome Atlas) database (https://portal.gdc.cancer.gov/), including mRNA seq level 3 data (*n* = 10,995), clinical data (*n* = 11,160), Illumina HumanMethylation 450k level 3 data (*n* = 10,129), SNV data (*n* = 10234), CNV data (*n* = 11,495), and miRNA transcript expression data (*n* = 9105). Reverse-phase protein array (RPPA) data (*n* = 7876) were obtained from the cancer proteome atlas (TCPA) database (https://tcpaportal.org/tcpa/index.html). TCPA RPPA data are all from TCGA samples across 32 cancer types. IC50 drug data of 481 small molecules in 1001 cell lines from the Therapeutics Response Portal (CTRP) (https://portals.broadinstitute.org/ctrp/) database were collected to investigate the correlation between gene expression of necroptosis-related regulators and drug sensitivity.

Totally, 30 GTEx normal tissues and 33 TCGA cancer types were included in the study. Normal tissue: adipose tissue, adrenal gland, bladder, blood, blood vessel, brain, breast, cervix uteri, colon, esophagus, fallopian tube, heart, kidney, liver, lung, muscle, nerve, ovary, pancreas, pituitary, prostate, salivary gland, skin, small intestine, spleen, stomach, testis, thyroid, uterus, vagina. Cancer type: Acute myeloid leukemia (LAML), adrenocortical carcinoma (ACC), bladder urothelial carcinoma (BLCA), breast invasive carcinoma (BRCA), cervical squamous cell carcinoma and endocervical adenocarcinoma (CESC), cholangiocarcinoma (CHOL), colon adenocarcinoma (COAD), esophageal carcinoma (ESCA), glioblastoma multiforme (GBM), head and neck squamous cell carcinoma (HNSC), kidney chromophobe (KICH), kidney renal clear cell carcinoma (KIRC), kidney renal papillary cell carcinoma (KIRP), lower grade glioma (LGG), liver hepatocellular carcinoma (LIHC), lung adenocarcinoma (LUAD), lung squamous cell carcinoma (LUSC), lymphoid neoplasm diffuse large B-cell lymphoma (DLBC), mesothelioma (MESO), ovarian serous cystadenocarcinoma (OV), pancreatic adenocarcinoma (PAAD), pheochromocytoma and paraganglioma (PCPG), prostate adenocarcinoma (PRAD), rectum adenocarcinoma (READ), sarcoma (SARC), skin cutaneous melanoma (SKCM), stomach adenocarcinoma (STAD), testicular germ cell tumors (TGCT), thymoma (THYM), thyroid carcinoma (THCA), uterine carcinosarcoma (UCS), uterine corpus endometrial carcinoma (UCEC), and uveal melanoma (UVM).

### Differential expression analysis in TCGA datasets

We used paired tumor and normal samples in the mRNA differential expression analysis to obtain more accurate results. A total of 14 cancer types (BLCA, BRCA, COAD, ESCA, HNSC, KICH, KIRC, KIRP, LIHC, LUAD, LUSC, PRAD, STAD, and THCA) were included in the final analysis, which had over 10 paired tumor and normal samples. RNAseq by Expectation-Maximization (RSEM) values were used to quantify the mRNA expression levels. The fold change (FC) was calculated by mean (Tumor)/mean (Normal), the *P*-value was estimated by *t*-test and was further adjusted by the FDR. Genes with the threshold of FC>2 and FDR ≤ 0.05 were considered as significantly differentially expressed.

### Subtype expression analysis and pathologic stage correlation

A great degree of intratumoral heterogeneity exists between tumors of different subtypes (molecular subtypes and clustering subtypes) in the same tumor type, which could be caused by different gene expression levels in different subtypes of tumors. We performed expression subtype analysis to identify subtype-relevant changes in gene expression. Nine cancer types (HNSC, LUSC, COAD, STAD, LUAD, GBM, BRCA, KIRC, and BLCA), which have at least 10 samples of each subgroup in a subtype, were included in the final analysis. The mRNA expression and clinical subtype data were merged by sample barcode. We compared the gene expression of necroptosis-related regulators among different subgroups in each subtype through the Wilcoxon test (number of subtype groups = 2) and ANOVA test (number of subtype groups > 2). Results were considered statistically significant at FDR ≤ 0.05. Furthermore, we performed trend analysis to explore the gene expression changes of necroptosis-related regulators with the progression of the clinicopathologic stage. Twenty-one cancer types with at least five samples in each stage subgroup were incorporated into the final analysis. The pathologic stage classified samples into main stages I, II, III, and IV.

### Survival analysis based on gene expression levels

We combined the mRNA expression data of necroptosis-related regulators and corresponding clinical survival data by sample barcode for expression survival analysis, and some uncensored data were left out. Based on the median RSEM value, we divided the tumor samples into high and low expression groups. Then, we used the R package “survival” to fit the survival status and survival time within the two groups. The Cox Proportional-Hazards model and log-rank test were used for every gene in every cancer. Genes with *P* ≤ 0.05 in Kaplan-Meier log-rank test were considered statistically significant.

### Methylation analysis

The 14 cancer types (THCA, KIRP, BLCA, LIHC, HNSC, BRCA, LUAD, PRAD, ESCA, KICH, LUSC, KIRC, STAD, COAD) having more than 10 samples both in tumor and adjacent non-tumor tissues were used to perform differential methylation analysis. The *P*-value was estimated by *t*-test and was further adjusted by FDR. Genes with FDR ≤ 0.05 were considered to have significant methylation differences.

Methylation can influence gene expression in theory. We combined the methylation and mRNA expression data via the TCGA barcode for correlation analysis between methylation levels and mRNA expression levels. Spearman correlation analysis was performed to identify the correlation between matched mRNA expression and methylation levels. The *P*-value was adjusted by FDR, and genes with FDR ≤ 0.05 were considered to be influenced significantly by genome methylation.

Methylation data and clinical overall survival data were merged by sample barcode. Similar to expression survival analysis, the tumor samples were divided into high and low methylation groups according to the median methylation level. The R package “survival” was used to fit survival time and survival status within the two groups. A Cox Proportional-Hazards model was constructed to determine the risk ratio (Hazard ratio) of the high methylation group compared with that of the low methylation group. The log-rank test was performed to test whether the survival difference between the two groups was statistically significant, and *P* ≤ 0.05 was considered significant.

### SNV analysis

Seven types of deleterious mutations were included in the SNV analysis: Missense_Mutation, Nonsense_Mutation, Frame_Shift_Ins, Splice_Site, Frame_Shift_Del, In_Frame_Del, and In_Frame_Ins. SNV summary and oncoplot waterfall plot were generated using maftools. The SNV percentage (frequency of deleterious mutations) of the coding region of each gene was calculated by the formula: Number of mutated samples/Number of cancer samples.

The SNV data and clinical survival data were merged by sample barcode. Tumor samples were divided into the mutant group when the specific gene was mutated (deleterious mutants). The log-rank test was performed to test the survival difference between wild-type (WT) and mutant groups.

### CNV analysis

In the CNV analysis, we calculated the percentage of CNV of each gene in each cancer type. The CNV could be divided into heterozygous CNV and homozygous CNV, including amplification and deletion. Heterozygous CNV represents the occurrence of CNV only on one chromosome, while homozygous CNV represents the occurrence of CNV on both chromosomes. The percentage statistic of CNV subtypes was based on CNV data processed through GISTICS 2.0.

We combined mRNA expression data and CNV raw data via TCGA barcode for correlation analysis. We calculated the association between matched mRNA expression and CNV percent samples based on Person’s product-moment correlation coefficient and t-distribution. *P*-value was adjusted by FDR.

The CNV data and clinical overall survival data were merged by sample barcode for survival analysis. The tumor samples were divided into WT, amplification, and deletion groups. The R package survival was used to fit survival time and survival status within groups. The log-rank test was performed to test the survival difference between the three groups, and *P* ≤ 0.05 was considered significant.

### MicroRNA (miRNA) regulation network analysis

mRNA expression data and miRNA expression data were combined by TCGA barcode. The miRNA regulation data were collected from experimentally verified data (TarBase, miRTarBase, and mir2disease) and predicted data (targetscan and miRanda), and only the miRNA-gene pairs that have been recorded in regulation data were used to calculate the expression correlation in all paired samples (33 cancers) based on Person’s product-moment correlation coefficient and t-distribution. In the presence of positive regulators like transcription factors, the miRNA-gene pair with negative correlation was considered a potential negatively regulation pair. *P*-value was adjusted by FDR, and genes with FDR ≤ 0.05 and R < 0 were used to generate the network via visNetwork R packages.

### Pathway activity analysis

The relative protein levels were obtained after TCPA RPPA data were median-centered and normalized by standard deviation across all samples for each component. The pathway score was then the sum of the relative protein levels of all positive regulatory components minus that of negative regulatory components in a particular pathway. We calculated the pathway activity score (PAS) of 10 famous cancer-related pathways (TSC/mTOR, RTK, RAS/MAPK, PI3K/AKT, Hormone ER, Hormone AR, EMT, DNA Damage Response, Cell Cycle, and Apoptosis pathways). Tumor samples were divided into two groups (high and low) by median mRNA expression, and the PAS difference between the two groups was determined by the Student *t*-test. When PAS (gene X high group) > (gene X low group), gene X was considered to activate the pathway; otherwise, it would inhibit the pathway. PAS with FDR ≤ 0.05 indicated a significant effect on the pathway.

### Immune subtype and drug sensitivity analysis

Immune subtype data were obtained from the UCSC Xena Browser (http://xena.ucsc.edu/). The correlation between mRNA expression and drug sensitivity was analyzed by merging all cancer cell lines’ mRNA expression and drug sensitivity data. Pearson correlation analysis was performed to determine the correlation between mRNA expression and drug IC50. *P*-value was adjusted by FDR. Only the top 30 ranked drugs were used to construct the plot after integrating the level of correlation coefficient and FDR of necroptosis-related regulators.

### GSVA

For GSVA, we estimated variation in gene set activity (represented as necroptosis score) over the cancer sample population in an unsupervised manner. The necroptosis score was calculated using the R package GSVA. It represented the integrated level of gene set expression, which was positively correlated with the expression of the gene set. The infiltrates of immune cells were evaluated using an ImmuCellAI web tool (http://bioinfo.life.hust.edu.cn/web/ImmuCellAI/). By integrating corresponding data and the necroptosis score, we further explored the crucial roles of necroptosis in the clinicopathologic stage, tumor subtype, prognosis, immune infiltration, and pathway regulation.

### Statistical analysis

All statistical analyses were performed using the R software v3.6 (http://www.r-project.org) and SPSS version 23.0 (SPSS Inc, Chicago, IL, USA).

### Data availability statement

The datasets presented in this study can be found in online repositories. The names of the repository/repositories and accession number(s) can be found in the article/Supplementary Material. Mapping data and analysis code are available at Supplementary Material.

## Supplementary Materials

Supplementary Material 1

Supplementary Material 2

Supplementary Material 3

Supplementary Material 4

Supplementary Material 5

Supplementary Material 6

Supplementary Material 7

Supplementary Material 8

Supplementary Material 9

Supplementary Material 10

Supplementary Material 11

Supplementary Material 12

Supplementary Material 13

Supplementary Material 14

Supplementary Material 15

Supplementary Material 16

Supplementary Material 17

Supplementary Material 18

Supplementary Material 19

Supplementary Material 20

Supplementary Material 21

Supplementary Material 22

Supplementary Material 23

Supplementary Material 24

Supplementary Figures
