# Supplementary material for: Multi-omics analysis reveals the panoramic picture of necroptosis-related regulators in pan-cancer
Source: Aging (Albany NY). 2022 Jun 21;14(12):5034–58. doi: 10.18632/aging.204124 (PMC9271292; doi:10.18632/aging.204124)
Supplement: Supplementary Figures [file aging-14-204124-s025.pdf]

SUPPLEMENTARY FIGURES

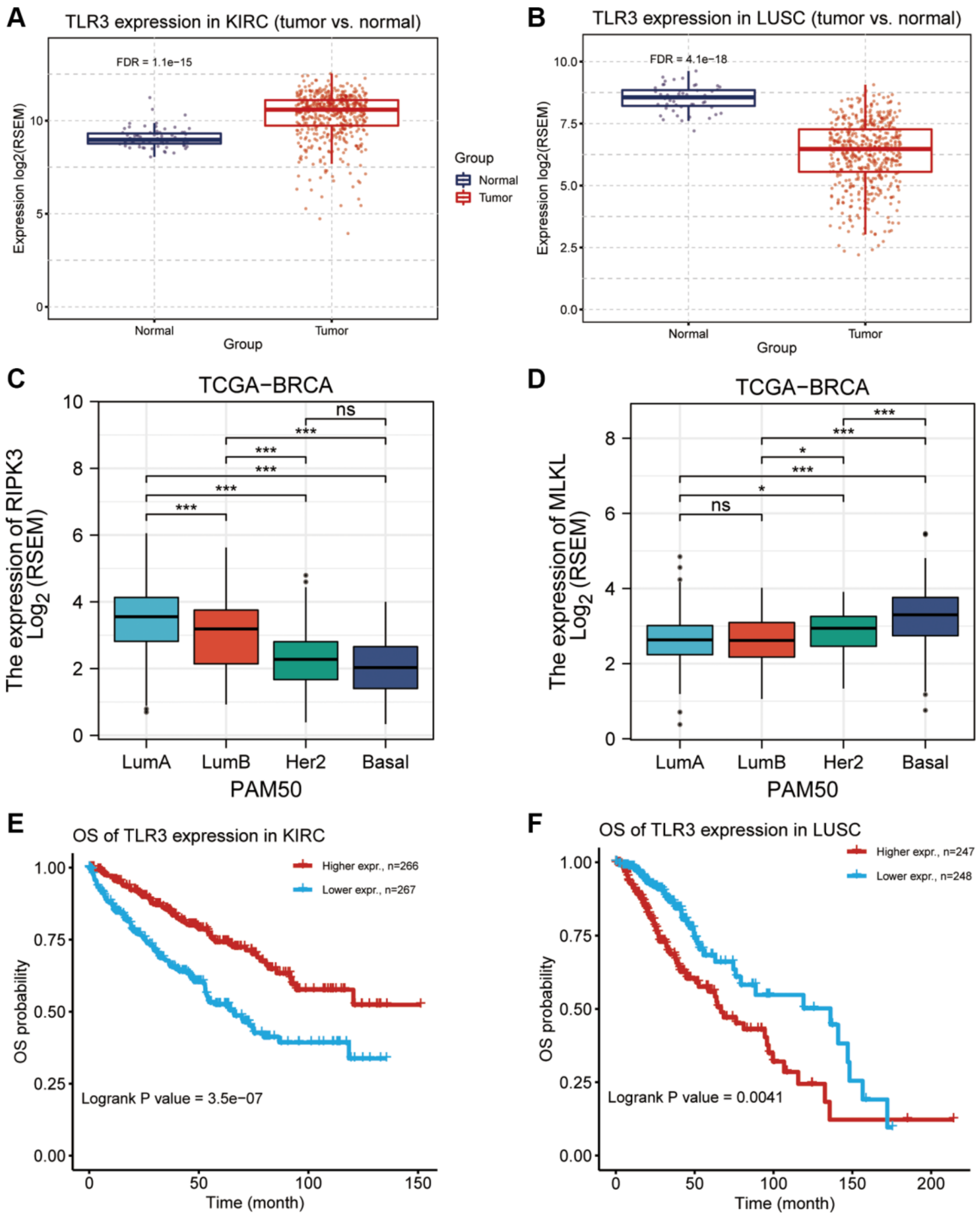

Supplementary Figure 1. (**A**) The differential expression of TLR3 between normal and tumor tissue in KIRC. (**B**) The differential expression of TLR3 between normal and tumor tissue in LUSC. (**C**) The expression of RIPK3 in different BRCA subtypes. (**D**) The expression of MLKL in different BRCA subtypes. (**E**) Kaplan-Meier curve between high and low expression of TLR3 in KIRC. (**F**) Kaplan-Meier curve between high and low expression of TLR3 in LUSC.

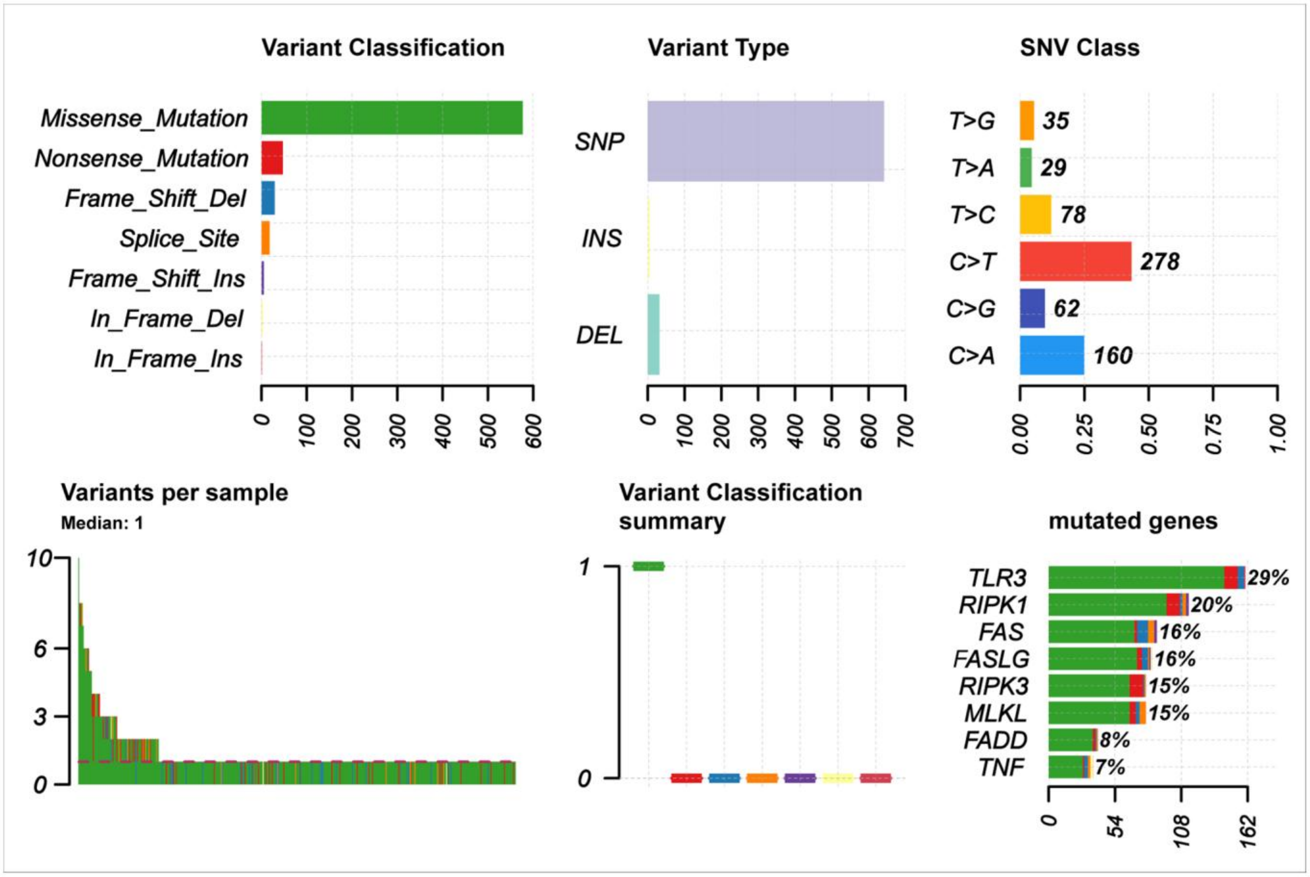

Supplementary Figure 2. SNV summary plot showing the number and types of mutations.

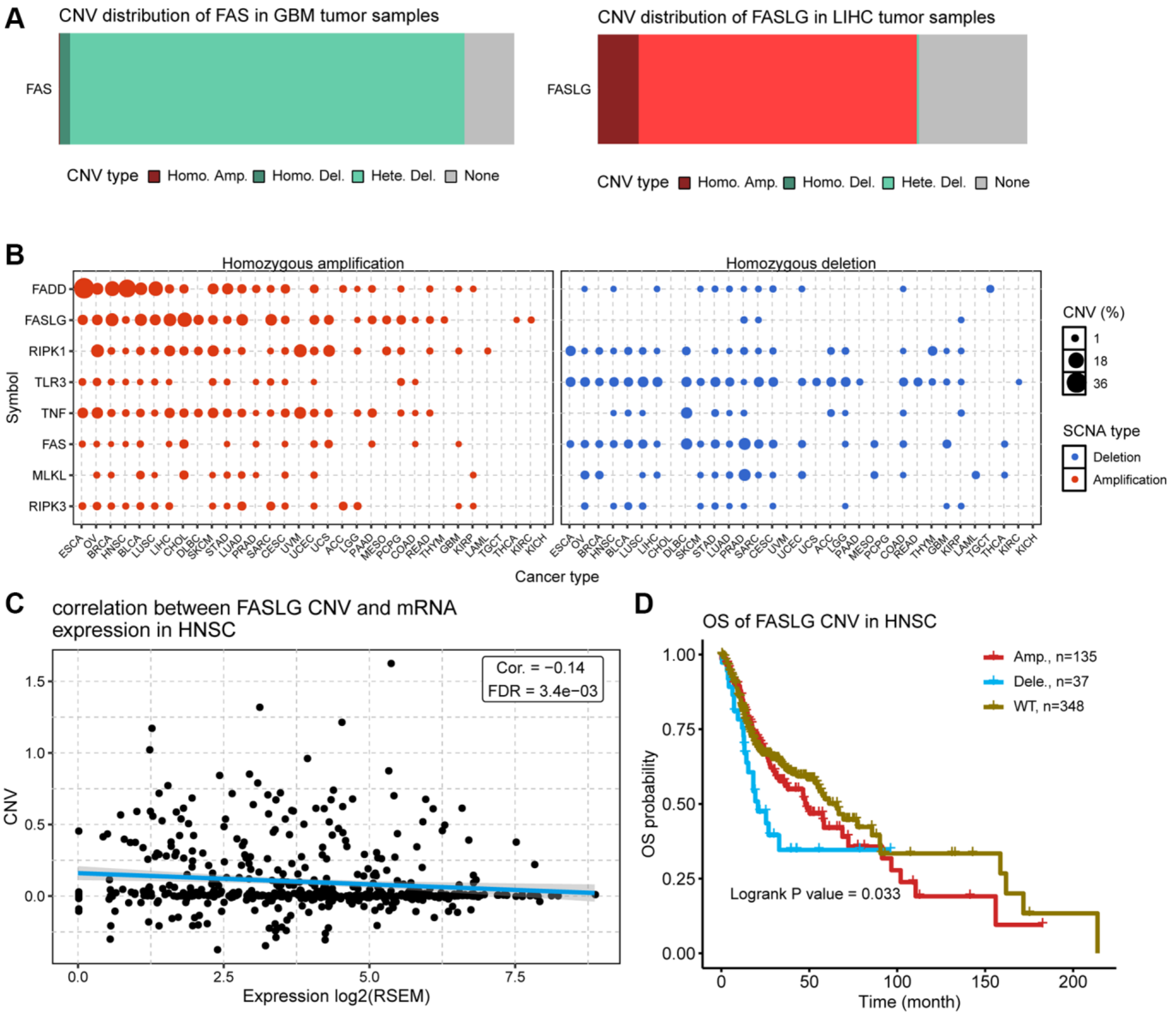

Supplementary Figure 3. (**A**) CNV type distribution of FAS in GBM and FASLG in LIHC. (**B**) Homozygous CNV plot showing the percentage of homozygous amplification and deletion of necroptosis-related regulators in different cancers. (**C**) Scatter plot showing the correlation between FASLG CNV and its mRNA expression in HNSC. (**D**) Kaplan-Meier curve showing the survival difference between different CNV types and wild type of FASLG in HNSC.

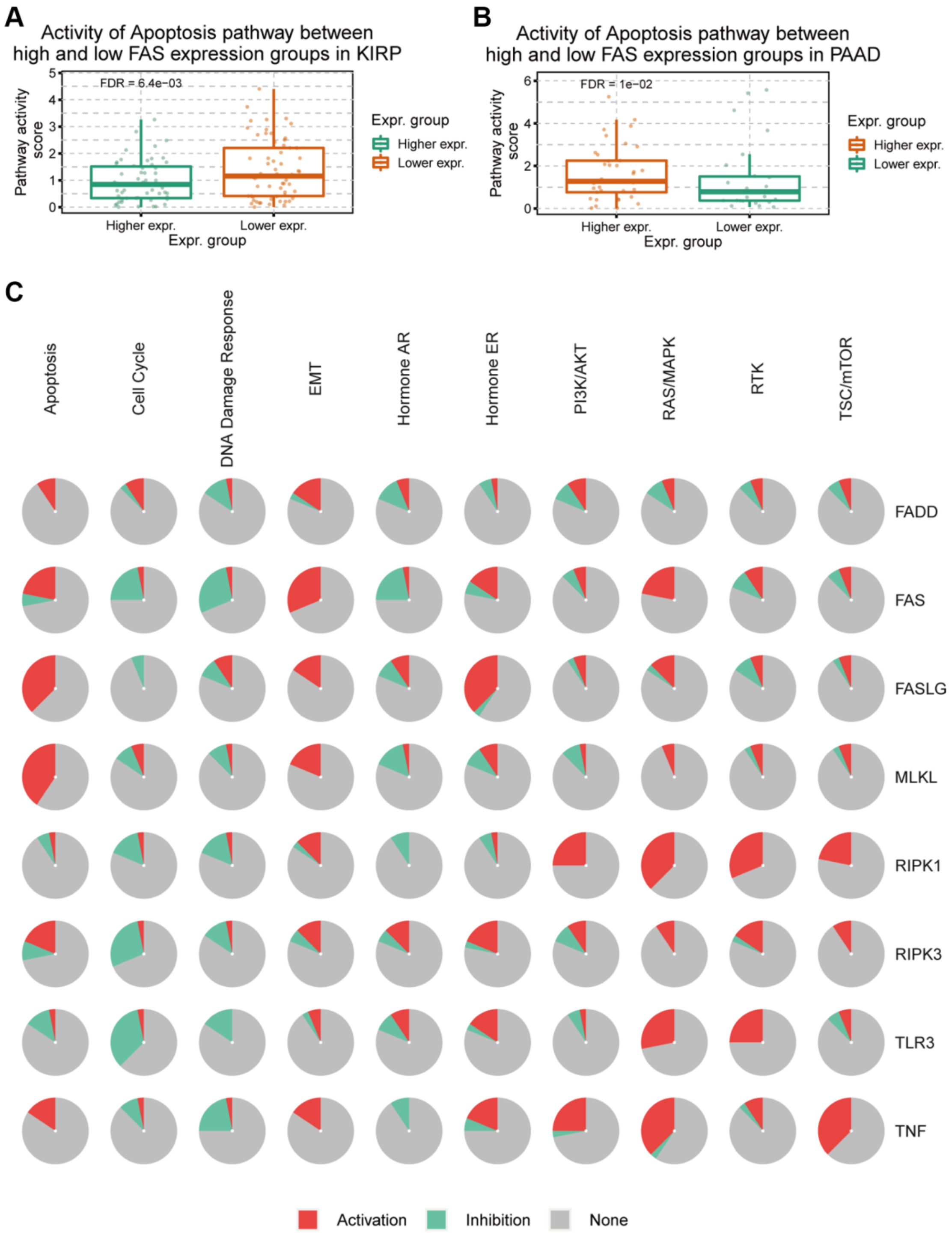

Supplementary Figure 4. (**A**) Box plot showing the difference of apoptosis pathway activity score between high and low FAS expression groups in KIRP. (**B**) Box plot showing the difference of apoptosis pathway activity score between high and low FAS expression groups in PAAD. (**C**) Pathway pie plot showing the global percentage of cancer types in which the specific necroptosis-related regulator has an effect on the specific pathway in pan-cancer.

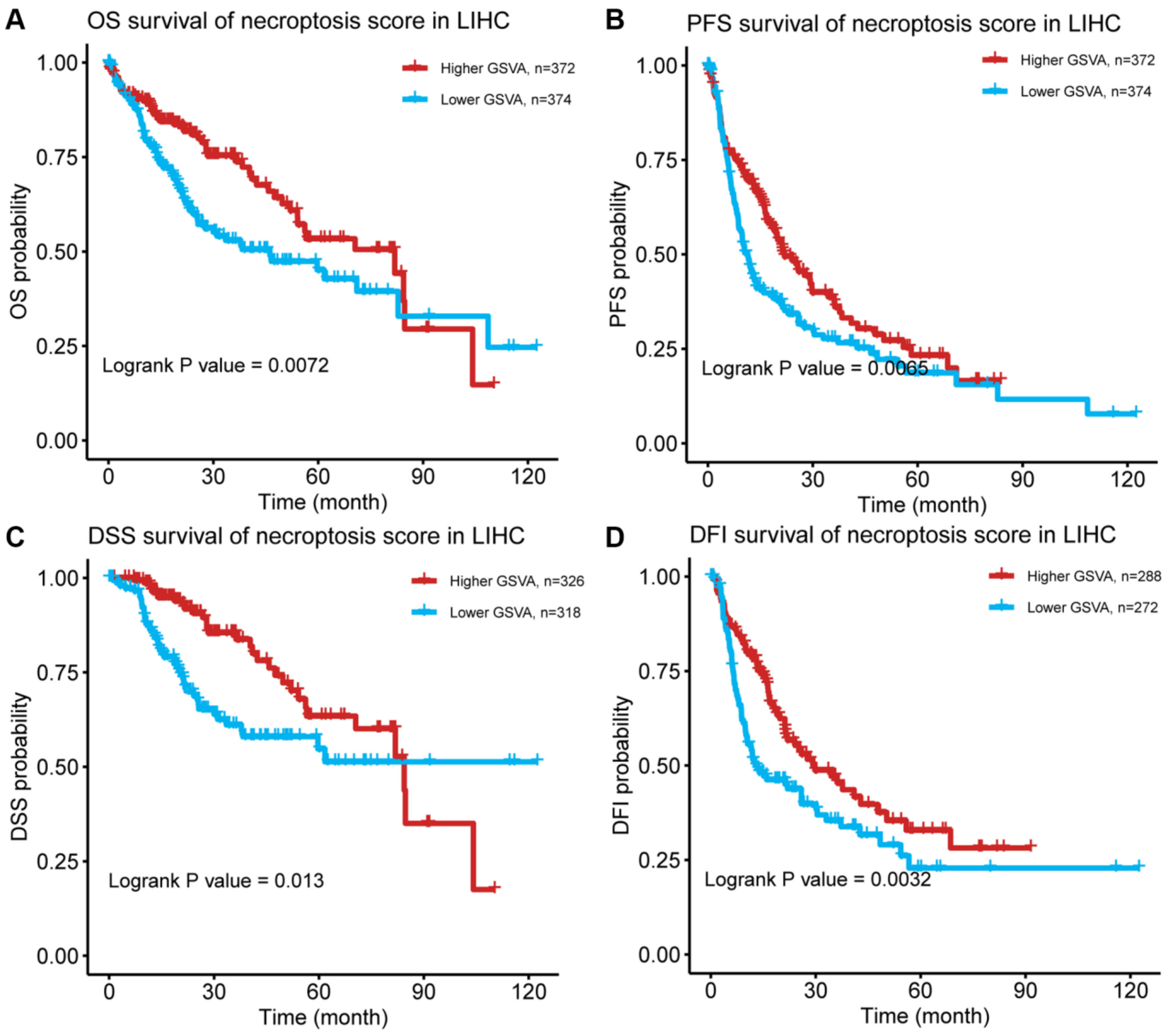

Supplementary Figure 5. Kaplan-Meier curve showing the survival difference between high and low necroptosis score in LIHC, including OS (**A**), PFS (**B**), DSS (**C**) and DFI (**D**).
